# STAT3 in acute myeloid leukemia facilitates natural killer cell-mediated surveillance

**DOI:** 10.3389/fimmu.2024.1374068

**Published:** 2024-07-05

**Authors:** Agnieszka Witalisz-Siepracka, Clio-Melina Denk, Bernhard Zdársky, Lorenz Hofmann, Sophie Edtmayer, Theresa Harm, Stefanie Weiss, Kerstin Heindl, Manuel Hessenberger, Sabrina Summer, Sayantanee Dutta, Emilio Casanova, Gerald J. Obermair, Balázs Győrffy, Eva Maria Putz, Heinz Sill, Dagmar Stoiber

**Affiliations:** ^1^ Division Pharmacology, Department of Pharmacology, Physiology and Microbiology, Karl Landsteiner University of Health Sciences, Krems, Austria; ^2^ Division Physiology, Department of Pharmacology, Physiology and Microbiology, Karl Landsteiner University of Health Sciences, Krems, Austria; ^3^ Department for Biomedical Research, University for Continuing Education Krems, Krems, Austria; ^4^ Division of Oncology, Medical University of Graz, Graz, Austria; ^5^ Institute of Pharmacology, Center of Physiology and Pharmacology & Comprehensive Cancer Center (CCC), Medical University of Vienna, Vienna, Austria; ^6^ Ludwig Boltzmann Institute for Hematology and Oncology, Medical University of Vienna, Vienna, Austria; ^7^ Department of Bioinformatics, Semmelweis University, Budapest, Hungary; ^8^ Department of Biophysics, Medical School, University of Pecs, Pecs, Hungary; ^9^ Cancer Biomarker Research Group, Institute of Molecular Life Sciences, HUN-REN Research Centre for Natural Sciences, Budapest, Hungary; ^10^ St. Anna Children’s Cancer Research Institute (CCRI), Vienna, Austria; ^11^ Institute of Pharmacology, Center of Physiology and Pharmacology, Medical University of Vienna, Vienna, Austria; ^12^ Division of Hematology, Medical University of Graz, Graz, Austria

**Keywords:** AML, NK cells, ICAM-1, immunotherapy, STAT3

## Abstract

Acute myeloid leukemia (AML) is a heterogenous disease characterized by the clonal expansion of myeloid progenitor cells. Despite recent advancements in the treatment of AML, relapse still remains a significant challenge, necessitating the development of innovative therapies to eliminate minimal residual disease. One promising approach to address these unmet clinical needs is natural killer (NK) cell immunotherapy. To implement such treatments effectively, it is vital to comprehend how AML cells escape the NK-cell surveillance. Signal transducer and activator of transcription 3 (STAT3), a component of the Janus kinase (JAK)-STAT signaling pathway, is well-known for its role in driving immune evasion in various cancer types. Nevertheless, the specific function of STAT3 in AML cell escape from NK cells has not been deeply investigated. In this study, we unravel a novel role of STAT3 in sensitizing AML cells to NK-cell surveillance. We demonstrate that STAT3-deficient AML cell lines are inefficiently eliminated by NK cells. Mechanistically, AML cells lacking STAT3 fail to form an immune synapse as efficiently as their wild-type counterparts due to significantly reduced surface expression of intercellular adhesion molecule 1 (ICAM-1). The impaired killing of STAT3-deficient cells can be rescued by ICAM-1 overexpression proving its central role in the observed phenotype. Importantly, analysis of our AML patient cohort revealed a positive correlation between *ICAM1* and *STAT3* expression suggesting a predominant role of STAT3 in ICAM-1 regulation in this disease. In line, high *ICAM1* expression correlates with better survival of AML patients underscoring the translational relevance of our findings. Taken together, our data unveil a novel role of STAT3 in preventing AML cells from escaping NK-cell surveillance and highlight the STAT3/ICAM-1 axis as a potential biomarker for NK-cell therapies in AML.

## 1 Introduction

Acute myeloid leukemia (AML) represents the second most common type of pediatric leukemia and the most common leukemia type in adults older than 50 years. In AML, heterogenous genetic alterations in blood cell progenitors result in overproduction of malignant myeloid precursors and stem cells (1). Five-year progression free survival of AML patients younger than 60 years lies between 35% and 40%, but drops dramatically in older patients (2, 3). The standard treatment for AML is chemotherapy, in certain cases followed by hematopoietic stem cell transplantation (1). Due to the identification of genetic alterations, novel targeted therapies have been developed in the last years. Nevertheless, relapse continues to be a significant concern affecting up to 50% of patients (4, 5), underlining the necessity to develop therapies focused on eliminating minimal residual disease (MRD). One emerging approach is natural killer (NK) cell immunotherapy, which holds the potential to address these unmet clinical needs (6, 7).

NK cells are innate lymphocytes that can recognize and kill malignant cells (8, 9). The sophisticated repertoire of activating and inhibitory receptors enables them to detect and efficiently eliminate cells that overexpress ligands for activating receptors or lack major histocompatibility complex (MHC) I molecules and therefore do not provide an inhibitory signal for NK cells (10, 11). Upon reaching the activation threshold, an immune synapse is formed at the NK cell-target cell contact (12, 13). The cytolytic granules containing perforin and granzymes polarize at the immune synapse which is a prerequisite for directed NK-cell degranulation and efficient elimination of the target cells (14, 15). This innate ability to lyse cancerous cells renders NK cells a powerful anti-leukemic tool. However, AML cells use different mechanisms to escape NK cell-mediated tumor surveillance. These include transcriptional downregulation and shedding of ligands for NK-cell activating receptors, upregulation of inhibitory ligands (16–19), as well as induction of immune suppressive signals derived from the microenvironment, such as indoleamine 2,3-dioxygenase or interleukin (IL)-10 (20). Downregulation of ligands for natural killer group 2 D (NKG2D), a crucial NK-cell activating receptor, by leukemic stem cells has been proven as an NK cell-escape mechanism in AML *in vivo* models (21). These observations were confirmed in the clinical setting, as low expression of the NKG2D ligand UL16 binding protein 1 (*ULBP1*) correlates with poor clinical outcome of AML patients (22).

Signal transducer and activator of transcription 3 (STAT3) is a member of the Janus kinase (JAK) – STAT pathway driving transcriptional responses downstream of many cytokines and growth factors including the IL-6 family (23). Besides its important role in regulating proliferation, survival and differentiation of cancer cells, STAT3 has a complex regulatory function in their immune evasion from NK-cell surveillance (23–25). Inhibition of STAT3 or upstream JAKs results in upregulation of NKG2D ligands and enhances NK cell-mediated lysis of different solid cancer cell lines (26–28). In AML however, the role of STAT3 in the regulation of NKG2D ligands or other NK-cell response-triggering molecules has not been thoroughly studied. Treatment of AML cell lines with rapamycin (29) or decitabine (30) leads to a downregulation of NKG2D ligands, which is associated with an increased activation of STAT3 but no causative relationship was demonstrated. On the other hand, STAT3 directly upregulates CD48, which is a surface molecule that enhances NK-mediated killing of AML cells suggesting an NK-sensitizing role of STAT3 in immune escape from NK cells (31, 32).

In the light of almost 40 ongoing clinical studies investigating NK cell-based immunotherapies in AML (*clinicaltrials.gov*, accessed on 18.04.2024), it is important to understand the mechanisms of AML escape from NK cells. We here show that deletion of STAT3 in the AML cell lines THP-1 and HEL leads to evasion from NK-cell recognition. Mechanistically, STAT3-deficient AML cells are hardly able to form an immune synapse due to decreased surface expression of intercellular adhesion molecule-1 (ICAM-1). *ICAM1* is of prognostic significance and shows a positive correlation with *STAT3* expression in our AML patient cohort. Our results unravel a novel role of STAT3 in preventing AML-cell escape from NK cells. In addition, we identify a STAT3/ICAM-1 axis as a potential biomarker for NK-cell therapies in AML.

## 2 Materials and methods

### 2.1 Mice and cell lines

NSG-Tg(Hu-IL15) (*NOD.Cg-Prkdc^scid^ Il2rg^tm1Wjl^ Tg(IL15)1Sz/SzJ*) mice (33) were purchased from the Jackson Laboratory and bred at the animal facility of the Medical University of Vienna (Himberg). Gender-matched and age-matched (8–12 weeks) animals were maintained under specific pathogen-free conditions according to FELASA recommendations (2014). All animal experiments were approved by the Ethics and Animal Welfare Committee of the Medical University of Vienna and granted by the national authority (Austrian Federal Ministry of Education, Science and Research) according to Section 8ff of the Law for Animal Experiments under license GZ BMBWF-66.009/0410-V/3b/2018 and were performed according to the guidelines of FELASA and ARRIVE.

The human AML cell lines THP-1 (34) and HEL (35) were purchased from DSMZ and cultured in RPMI1640 complete medium (Gibco) supplemented with L-glutamine (Gibco), 10% fetal bovine serum (FBS) (Thermo Fisher Scientific), 50 µM 2-mercaptoethanol, 100 U/mL penicillin and 100 µg/mL streptomycin (Gibco). The human NK cell lines NK92 (36) and KHYG1 (37); purchased from ATCC; were cultured in RPMI1640 supplemented with L-glutamine (Gibco), 20% FBS (Thermo Fisher Scientific), 50 µM 2-mercaptoethanol, 100 U/mL penicillin and 100 µg/mL streptomycin, 10 mM HEPES (Gibco), non-essential amino acids (Gibco) and 20 ng/mL of human recombinant (rh)IL-2 (ImmunoTools). The cell lines were routinely tested for mycoplasma contamination.

To obtain STAT3-deficient AML cell lines, HEL and THP-1 cells were lentivirally transduced with particles containing lentiCRISPR v2 (Addgene #52961) with respective sgRNAs. The following sgRNA oligonucleotides were used: NT-sgRNA: 5’-CACCGACGGAGGCTAAGCGTCGCAA-’3; STAT3-sgRNA6: 5’-CACCGCATTCGACTCTTGCAGGAAG-’3; STAT3-sgRNA8: 5’-CACCGCAGCTTGACACACGGTACC-’3. Transduced cells were treated with 2-4 µg/mL puromycin (Sigma-Aldrich) and single cell-derived clones were acquired via serial dilution.

To obtain ICAM-1 overexpressing cell lines, THP-1 and HEL STAT3^WT^ and STAT3^KO^ cell lines were lentivirally transduced with particles containing *pHIV-EGFP* (Addgene # 21373) or *pHIV-ICAM1-EGFP*. In brief, the coding sequence of *ICAM1* was obtained by RNA isolation from THP-1 STAT3^WT^ cells using RNeasy Mini Kit (Qiagen) followed by reverse transcription using random primers and RevertAid H Minus First Strand cDNA Synthesis Kit (Thermo Fisher Scientific). The gene of interest was cloned with *ICAM1*-specific primers and ligated into *pHIV-EGFP* vector. Transduced cell lines were used for experiments when >95% of cells were GFP^+^.

### 2.2 Primary human NK cell isolation

The study was granted approval by the Scientific Integrity and Ethics Committee at Karl Landsteiner University of Health Sciences in Krems, Austria. Healthy donor human NK cells (hNK) were isolated from *Leukocyte Reduction System* chambers (Transfusion Medicine and Blood Group Serology, University Hospital Vienna; ethics number) using RosetteSep Human NK cell Enrichment Cocktail (Stem Cell Technologies) according to the manufacturer’s instructions. NK cells (CD3^−^CD56^+^ >90%) were expanded in basal NK cell MACS medium (Miltenyi Biotec) supplemented with 5% FBS, 100 U/mL penicillin and 100 µg/mL streptomycin, 1% NK cell MACS medium supplement (Miltenyi Biotec) and 20 ng/mL (rh)IL-2 (ImmunoTools).

### 2.3 *In vivo* AML mouse model

NSG-Tg(Hu-IL15) mice were randomly assigned into two groups, intravenously (*i.v.*) injected either with 10^6^ primary NK cells expanded for 24 h as described above or left untreated. One day later mice were injected *i.v.* with 0.5x10^6^ HEL or THP-1 cells. The bone marrow and spleens were isolated 28 days post AML cell injection and single cell suspensions were analyzed via flow cytometry. For survival analysis the disease progression was monitored in a blinded manner and the mice were euthanized at the first signs of the disease. Bone marrow and spleens were isolated and single cell suspension cryopreserved.

### 2.4 AML patient samples

The study was granted approval by the Scientific Integrity and Ethics Committee at Karl Landsteiner University of Health Sciences in Krems, Austria, and adhered to the principles of the Declaration of Helsinki. We obtained samples from 79 adult individuals diagnosed with AML (with no FLT3 aberration; in accordance with the guidelines provided by the European LeukemiaNet in 2022 (38)) upon written informed consent at the Medical University of Graz (Graz, Austria). Patients’ characteristics are available in **Supplementary Table S1**. Mononuclear cells from peripheral blood or bone marrow aspirates, were isolated using Ficoll density gradient (GE Healthcare), as previously described (39). Subsequently, the cells were cryopreserved in FBS (Gibco, Thermo Fisher Scientific) containing 10% dimethyl sulfoxide (Sigma Aldrich). In addition to our collected data, we integrated a publicly available dataset accessed via kmplot.com (40), with the latest update as of September 13, 2023. Uni- and multivariate Cox regression analysis was performed to correlate survival with *ICAM1* expression, age, gender and karyotype (good, intermediate, poor). The multivariate analysis was performed by pairing *ICAM1* with the clinical variables. The analysis was performed in pairs to maximize the sample number available for the analysis. All AML patients with no FLT3 aberrations available in the kmplot.com database were included in the analysis.

We have also performed correlation analysis on normalized gene expression RNA-seq data of human AML patients from The Cancer Genome Atlas (TCGA-LAML). The correlation coefficients between any gene and *ICAM1* were calculated using the Spearman correlation. The ranked gene list based on the correlation coefficient with *ICAM1* gene expression was used for gene set enrichment analysis (GSEA version 4.3.3) which was carried out according to the provider’s protocol (41). Gene sets were downloaded from the Molecular Signature Database (http://software.broadinstitute.org/gsea/msigdb/index.jsp) and permutations were set to 1,000.

### 2.5 Western blot

For Western blot analysis 10^6^ cells were lysed directly in 100 µl of 1 × Laemmli sample buffer (Bio-rad). SDS-PAGE and Western blots were performed as previously described (42). The following antibodies were purchased from Cell Signaling Technology: pSTAT3 (Tyr705, D3A7), STAT3 (D3Z2G), anti-β-ACTIN (#4967), anti-rabbit IgG HRP-linked (#7074P2).

### 2.6 RNA isolation and qPCR

RNA was isolated using QIAzol Lysis Reagent (Qiagen) or RNeasy Mini Kit (Qiagen; patient samples). Reverse transcription was performed using RevertAid H Minus First Strand cDNA Synthesis Kit (Thermo Fisher Scientific) according to the manufacturer’s instructions. Quantitative real time PCR (qPCR) using SsoAdvanced™ Universal SYBR^®^ Green Supermix (Bio-rad) was analyzed in duplicates on a qTower^3^ (Analytik Jena). *GAPDH* was used as internal control and the relative expression was quantified as described previously (43). The fold change of expression in STAT3-deficient cell lines was calculated as relative to the respective control cell line. The ratio of expression between each replicate and mean of STAT3^WT^ samples was calculated for each experiment. Primer sequences are listed in **Supplementary Table S2**.

### 2.7 Flow cytometry

THP-1 or HEL cells were incubated with FcX blocking antibody (Biolegend) followed by staining with ICAM-1 (HA58, BD Biosciences), HLA-A/B/C (W6/32, Biolegend), CD48 (BJ40, Biolegend), CD58 (TS2/9, Biolegend), ULBP2/5/6 (165903, BD Biosciences), B7-H6 (1A9, BD Biosciences) MICA/B (6D4, Thermo Fisher Scientific) antibodies. Single cell suspensions from spleen or bone marrow of leukemic mice were incubated with FcX blocking antibody (Biolegend) and stained for human CD45 (HI30, Biolegend) and human NKp46 (9E2, Biolegend). Flow cytometry analysis was performed on Cytoflex S (Beckman Coulter) or Cytoflex LX (Beckman Coulter) and analyzed on CytExpert software V. 2.4 (Beckman Coulter).

### 2.8 *In vitro* cytotoxicity assays

Expanded NK cells, NK92 or KHYG1 cell lines were co-cultured with 10^4^ CFSE-stained (1 µM) target AML cells at different effector-to-target (E:T) ratios in duplicates. After 4 h of co-incubation all samples were subjected to SYTOX™ Blue Dead Cell Stain (Thermo Fisher Scientific) for 1 min and analyzed by flow cytometry. The specific lysis was calculated by subtracting percentage of Sytox^+^CFSE^+^ cells incubated without NK cells from percentage of Sytox^+^CFSE^+^ cells incubated with NK cells. For the ICAM-1 blocking experiment, AML cells were incubated with mouse IgG1κ (isotype control; MOPC-21, Biolegend) or purified anti-CD54 antibody (HCD54, Biolegend) for 45 min at 4°C prior to co-incubation with NK92 cells.

### 2.9 Degranulation assay

5x10^4^ CFSE-stained (1 µM) THP-1 or HEL cells were seeded in 96-well plates and co-incubated with 5x10^4^ human NK cells from three different donors for 2 h at 37°C in the presence of anti-CD107a (H4A3, Biolegend) antibody and Brefeldin A (Biolegend). The expression of CD107a on NK cells (CFSE^-^ - in contrast to CFSE^+^ AML cells) was analyzed by flow cytometry.

### 2.10 IFNγ intracellular staining

10^5^ CFSE-stained (1 µM) THP-1 or HEL cells were seeded in 5 ml tubes and co-incubated with 10^5^ NK92 cells for 4 h at 37°C in the presence of Brefeldin A (Biolegend). The cells were fixed and permeabilized using the eBioscience™ Foxp3/Transcription Factor Staining Buffer Set according to the manufacturer’s instructions (Thermo Fisher Scientific). The cells were stained for 30 min with anti-IFNγ antibody (B27; ImmunoTools). The expression of IFNγ in NK92 cells (CFSE^-^ - in contrast to CFSE^+^ AML cells) was analyzed by flow cytometry.

### 2.11 Conjugate formation assay

NK92 or KHYG1 cells were stained with CellTrace™ Violet Cell Proliferation Kit (Thermo Fisher Scientific) and AML target cell lines were stained with CellTrace™ CFSE Cell Proliferation Kit (Thermo Fisher Scientific) according to the manufacturer’s protocol. NK92 or KHYG1 cells were mixed with AML cells at a 1:1 ratio in cold RPMI1640 complete medium. Conjugate formation was induced by incubation at 37°C for 5, 10 or 20 min and then stopped by adding ice-cold PBS. The percentage of doublets double positive for CellTrace Violet and CFSE was analyzed by flow cytometry. For fluorescence microscopy, NK92 cells were additionally stained with anti-CD56-PE antibody (MEM-188, Biolegend). The conjugates were immobilized on poly-L-lysine (Sigma) coated cover slips, fixed with 4% paraformaldehyde (Fisher Bioreagents) and imaged using Olympus BX53 fluorescent microscope (20x, 0.85 N.A. objective) and a XM10 (Olympus) cooled CCD camera. The pictures were analyzed using the OLYMPUS cellSens Dimension software (Olympus). Quantification of synapse area was performed by calculating the ratio of mean fluorescence intensity of CD56 at the NK92-THP-1 contact site to mean fluorescence intensity of CD56 at total NK92 surface using ImageJ software.

### 2.12 Statistical analysis

Unpaired t-tests or one-way ANOVA with Tukey post-tests were conducted using GraphPad Prism^®^ version 9.00 or 10.00 (GraphPad Software). For correlation analysis, Pearson correlation coefficient was calculated and a line was fitted using simple linear regression. Kaplan-Meier estimates were utilized for survival analysis, with differences assessed through log-rank (Mantel-Cox) tests. Both analyses were performed using GraphPad Prism^®^ version 9.00 or 10.00 (GraphPad Software). The significance level is indicated for each experiment as follows: (*p < 0.05; ** p < 0.01; ***p < 0.001; ****p < 0.001). Only significant differences are indicated. Outliers were identified using Outlier Calculator (GraphPad Software). For patient sample gene expression analysis, the patient cohort was stratified into subgroups with high (relative expression > 0.1) and low (relative expression < 0.1) *STAT3* expression or high (relative expression > 0.28) and low (relative expression <0.28) *ICAM1* expression as previously described (44).

## 3 Results

### 3.1 THP-1 and HEL cell lines are killed by NK cells *in vivo*

**Figure 1:**
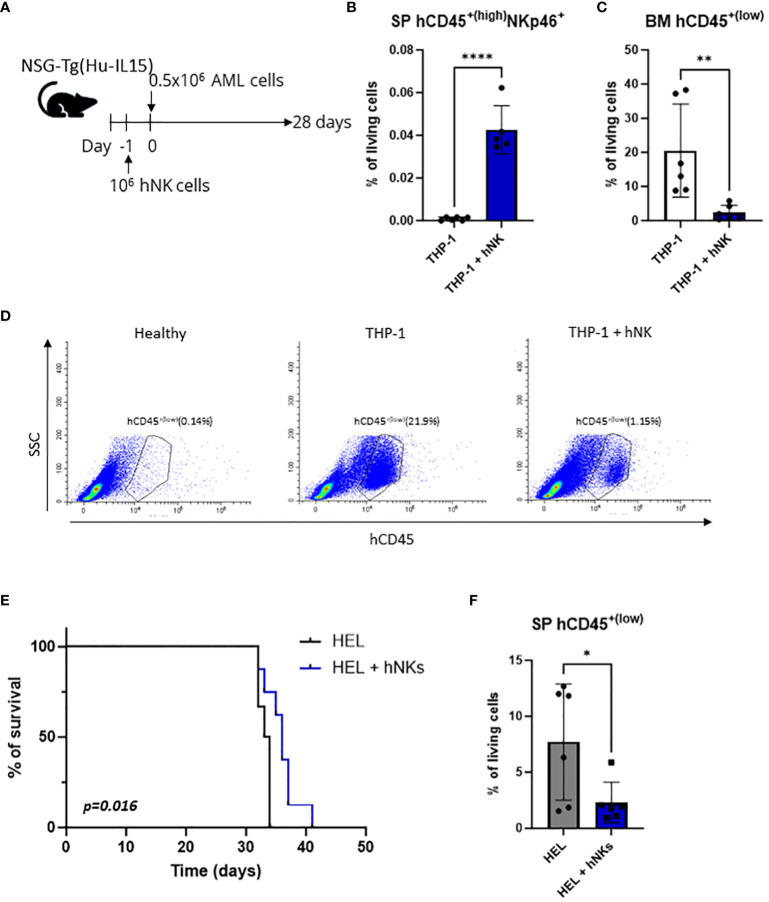
THP-1 and HEL cell line xenografts are killed by NK cells *in vivo*. **(A–D)** NSG-Tg(Hu-IL15) mice were injected via the tail vein with 0.5x10^6^ THP-1 cells. In addition, the mice were injected *i.v.* with 10^6^ human NK cells 24 h prior to leukemic cell inoculation or left untreated. **(A)** Schematic experimental setup. **(B)** hCD45^+(high)^ NKp46^+^ NK cells were analyzed by flow cytometry in the splenic single cell suspensions 28 days post injection. **(C, D)** Infiltration of hCD45^+(low)^ leukemic cells in the bone marrow 28 days post injection was analyzed by flow cytometry. **(B, C)** Bar graphs represent mean +/- SD from two independent experiments (n=5-6 per group). Statistical analysis was performed using unpaired t-test. **(D)** Representative flow cytometry plots. **(E, F)** NSG-Tg(Hu-IL15) mice were injected with 0.5x10^6^ HEL cells *i.v*. In addition, the mice were injected *i.v.* with 10^6^ human NK cells 24 h prior to leukemic cell inoculation or left untreated. **(E)** Kaplan-Meier plot showing survival of mice injected with HEL cells only (n=6) or additionally with NK cells (n=8) as pool of two independent experiments. Kaplan-Meier estimates were utilized for survival analysis, with differences assessed through log-rank (Mantel-Cox) test. **(F)** hCD45^+(low)^ leukemic cells were analyzed by flow cytometry in the splenic single cell suspensions of terminally diseased animals. Bar graphs represent mean +/- SD from two independent experiments (n=6 per group). Statistical analysis was performed using unpaired t-test. **(B, C, F)** *p < 0.05; **p<0.01; ****p<0.0001.

NK cell-adoptive transfer is one of the most promising NK cell-based therapeutic approaches for AML. To test whether the AML cell lines used in this study are controlled by adoptively transferred NK cells, we established a humanized xenograft model. NSG-Tg (Hu-IL15) mice were injected with human NK cells prior to intravenous transplantation of the AML cell lines HEL or THP-1 (**Figure 1A**). The HEL cell line is of an erythroleukemic subtype and has not been widely used as an NK-cell target, while the THP-1 cell line belongs to the acute monocytic leukemia subtype that was shown to be efficiently killed by NK cells *in vitro* (45).

The engraftment of NK cells (hCD45^+(high)^ NKp46^+^) was confirmed in the spleen of THP-1 (**Figure 1B**, **Supplementary Figure S1A**) and HEL (**Supplementary Figure S1B**) injected mice 28 days post injection. On the same day infiltration of hCD45^+(low)^ THP-1 cells in the bone marrow of control mice was detected, while barely any THP-1 cells were present in the NK cell-treated mice (**Figures 1C, D**). Similarly, but less pronounced, NK-cell injection resulted in decreased infiltration of hCD45^+(low)^ HEL cells into the bone marrow (**Supplementary Figure S1C**). To test whether this minor effect in the bone marrow infiltration of HEL cells translates into a survival benefit we used the same approach (**Figure 1A**) but monitored the disease onset over longer time. Mice injected only with the HEL cell line succumbed to the disease after 35 days and showed the mean of 7% leukemic cell infiltration in the spleen (**Figures 1E, F**). Mice which received primary human NK cells prior to HEL cell injection displayed an increased disease latency and on average 2% of leukemic cells in the spleen (**Figures 1E, F**), while at the timepoint of euthanasia the leukemic cells infiltrated the bone marrow to a similar extent (**Supplementary Figure S1D**). NK cells were also detectable in the spleen of leukemic animals at the experimental endpoint (**Supplementary Figure S1E**). These data demonstrate the susceptibility of two human AML cell lines (THP-1 and HEL) to *in vivo* killing by adoptively transferred NK cells in an AML xenograft model using NSG-Tg (Hu-IL15) mice. Therefore, these cell lines proved as a useful tool to study NK-cell surveillance of AML in the context of NK cell-based immunotherapies.

### 3.2 STAT3-deficient AML cells escape NK-cell recognition

**Figure 2:**
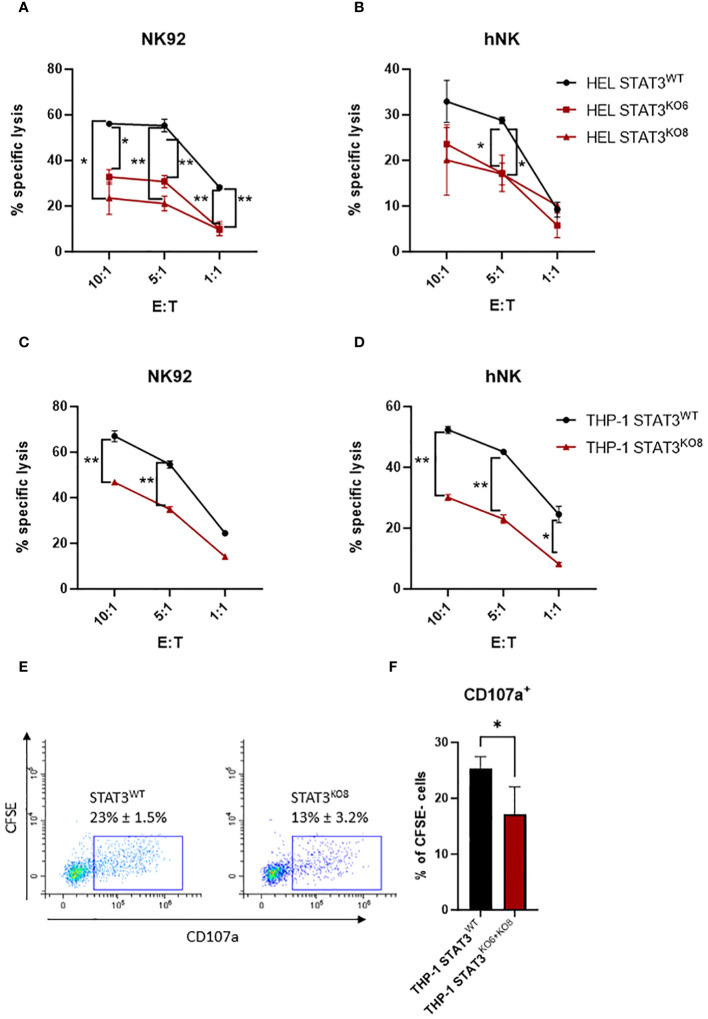
STAT3-deficient AML cells escape NK-cell recognition. **(A, B)** CFSE-stained HEL STAT3^WT^, STAT3^KO6^ and STAT3^KO8^ cells were mixed at indicated effector: target (E:T) ratios with **(A)** NK92 for 4 h or **(B)** expanded primary human NK cells for 2 h. The specific lysis of target cells was assessed by flow cytometry. One representative out of at least two independent experiments is shown. **(C, D)** CFSE-stained THP-1 STAT3^WT^ and STAT3^KO8^ cells were mixed at indicated effector: target (E:T) ratios with **(C)** NK92 for 4 h or **(D)** expanded primary human NK cells for 2 **(h)** The specific lysis of target cells was assessed by flow cytometry. One representative out of at least two independent experiments is shown. **(A–D)** Symbols and bars represent mean of technical duplicates +/- SD. Statistical analysis for each ratio was performed using **(A, B)** one-way ANOVA with Tukey post-test (STAT3^WT^ vs ^KO6^ – indicated on the left; STAT3^WT^ vs ^KO8^ indicated on the right side) or **(C, D)** unpaired t-test. **(E, F)** CFSE stained THP-1 STAT3^WT^ and STAT3^KO6^ and STAT3^KO8^ cells were mixed at 1:1 ratio with human primary NK cells from three different healthy donors and the percentage of CD107a^+^ NK cells (CFSE-; in contrast to CFSE+ target cells) was analyzed by flow cytometry. **(E)** Representative flow cytometry plots pre-gated on CFSE- cells (NK cells only). **(F)** Bar graphs represent mean +/- SD of n= 4 per group (3 donors; each donor 1-2 experiments). Statistical analysis was performed using unpaired t-test. **(A–D, F)** *p < 0.05, **p < 0.01.

To investigate the impact of STAT3 on the susceptibility of AML cells to NK-cell lysis, we generated STAT3-deficient HEL cell lines using CRISPR-Cas9. Both sgRNAs targeting STAT3 led to an efficient knockout of STAT3 (STAT3^KO6^ and STAT3^KO8^) compared to non-targeting control sgRNA (STAT3^WT^) (**Supplementary Figure S2A**). Upon 4 h incubation with the human NK cell line NK92, the HEL control cell line was killed efficiently while the killing of both STAT3-deficient cell lines was significantly reduced (**Figure 2A**). To confirm our results, we isolated human primary NK cells from healthy donors and analyzed their responses to HEL STAT3^WT^ and HEL STAT3^KO^ cell lines. Although the degranulation capacity of human primary NK cells was not affected by STAT3 deficiency in HEL cells (**Supplementary Figure S2B**), the cytotoxic capacity of primary NK cells was impaired towards the STAT3-deficient HEL cells compared to the STAT3^WT^ cells (**Figure 2B**).

To further expand our findings to another AML subtype, we generated STAT3-deficient THP-1 cells using the same approach as for HEL cells (**Supplementary Figure S2A**). In line with our previous observations, THP-1 cells expressing STAT3 were killed efficiently by NK92 cells and deletion of STAT3 resulted in a reduction of the killing capacity (**Figure 2C** - STAT3^KO8^, **Supplementary Figure S2C** - STAT3^KO6^). Likewise, another cytotoxic NK cell line, KHYG1, showed strongly impaired killing capacity of STAT3-deficient THP-1 cells (**Supplementary Figure S2D**). Also, primary NK cells killed STAT3-deficient THP-1 cell lines less efficiently compared to the STAT3^WT^ cells (**Figure 2D** - STAT3^KO8^; **Supplementary Figure S2E** - STAT3^KO6^). In line, staining of primary NK cells with anti-CD107a antibody showed a significant reduction of degranulating NK cells upon incubation with STAT3-deficient THP-1 cells compared to control cells (**Figures 2E, F**). Interestingly, also IFNγ production was impaired in NK92 cells incubated with THP-1 STAT3^KO^ cells while HEL cells induced barely any IFNγ production under tested experimental conditions (**Supplementary Figure S2F**). Taken together, STAT3-deficient AML cells escape the killing by human NK cell lines and primary NK cells.

### 3.3 STAT3-deficient AML cells show reduced expression of ICAM-1

**Figure 3:**
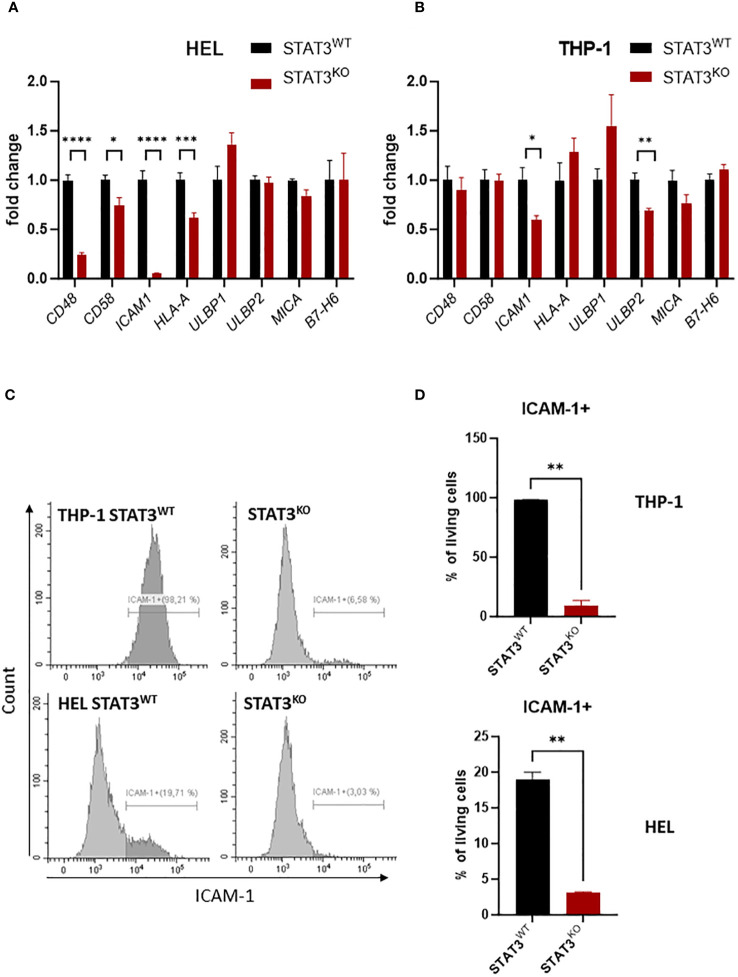
STAT3-deficient AML cells show reduced expression of ICAM-1 **(A, B)** Expression levels of *CD48, CD58, ICAM1, HLA-A, ULBP1, ULBP2*, *MICA, B7-H6* were analyzed in **(A)** HEL and **(B)** THP-1 STAT3^WT^ and STAT3^KO8^ cells using RT-qPCR (n=6-8 per group, out of two or three independent experiments). The relative values to the respective control cell line are shown as mean +/- SEM in the bar graphs. Statistical analysis was performed using unpaired t-test for each gene. **(C, D)** THP-1 and HEL STAT3^WT^ and STAT3^KO8^ were analyzed for surface expression of ICAM-1 via flow cytometry. Representative histograms **(C)** and bar graphs showing mean percentage of ICAM-1^+^ cells +/- SD **(D)** are shown for each cell line (n=2 per group from two independent experiments). Statistical analysis was performed using unpaired t-test. **(A, B, D)** *p < 0.05, **p < 0.01, ***p < 0.001, ****p < 0.0001.

To investigate the mechanisms underlying the impaired killing by NK cells in the absence of STAT3, we screened the mRNA expression of known NK-cell activating and inhibitory ligands in control and STAT3-deficient THP-1 and HEL cell lines via RT-qPCR. As both STAT3-deficient cell lines were similarly sensitive to NK-cell killing, only the STAT3^KO8^ from each cell line was used for further experiments (referred to further on as STAT3^KO^). We observed that in both cell lines the activating NKp30 ligand *B7-H6* and the NKG2D ligands *ULBP1* and *MICA* did not show any significant STAT3-dependent regulation, while *ULBP2* showed a downregulation only in the THP-1 cell line (**Figures 3A, B**). In the absence of STAT3, molecules involved in NK-cell adhesion to target cells such as *CD48* and *CD58* were significantly lower expressed in HEL cells (**Figure 3A**), while *ICAM1* was strongly downregulated in both cell lines (**Figures 3A, B**). Further, we validated that in HEL cells CD48, but not CD58 surface protein expression was mildly decreased in the absence of STAT3 and opposite effect was observed in THP-1 cells (**Supplementary Figures S3A, B**). MHC I molecules are the most important inhibitory ligands for NK cells (46). Although *HLA-A* mRNA expression was downregulated in STAT3^KO^ HEL cells (**Figure 3A**), MHC I protein expression (HLA-A/B/C) was not affected by the lack of STAT3 in any of the investigated cell lines (**Supplementary Figure S3C**). Surface expression of NKG2D ligands ULBP2/5/6 and MICA/B was decreased in STAT3^KO^ THP-1 cells but remained unaffected in HEL cells (**Supplementary Figures S3D, E**), while STAT3 had no effect on B7-H6 levels in any of the cell lines (**Supplementary Figure S3F**). In line with gene expression data in both cell lines, ICAM-1 was hardly detectable on the surface of the cells lacking STAT3, while a higher percentage of control cells was ICAM-1 positive (**Figures 3C, D**). Thus, ICAM-1 deregulation is a common feature of the studied STAT3-deficient AML cells. In summary, STAT3-deficiency in AML cells results in decreased killing by NK cells and strongly reduced ICAM-1 surface expression.

### 3.4 STAT3-deficient AML cells form the immune synapse with NK cells less efficiently

**Figure 4:**
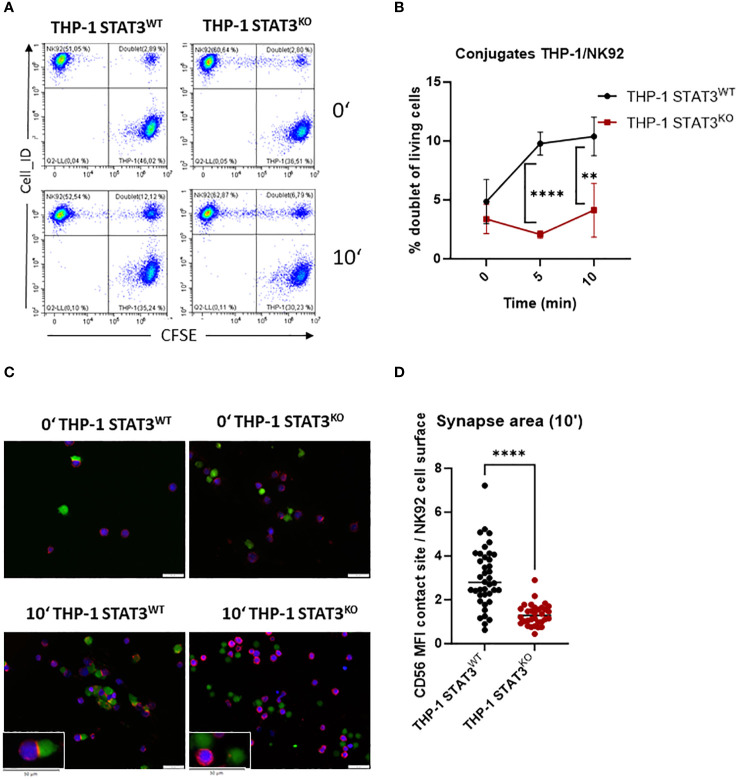
STAT3-deficient AML cells form fewer immune synapses with NK cells. **(A, B)** THP-1 STAT3^WT^ and STAT3^KO8^ were incubated with NK92 at 1:1 ratio and the number of NK92-THP-1 doublets was analyzed after indicated timepoints. **(A)** Representative flow cytometry plots. **(B)** Symbols and bars represent the mean percentage of doublets +/- SD (n=3-4 per group from 2 independent experiments). Statistical analysis was performed using unpaired t-test for each timepoint. **(C)** Representative fluorescence microscopy pictures showing THP-1 cells in green and NK92 in blue and stained for CD56 in red. **(D)** The synapse area from **(C)** was quantified for timepoint 10 min as ratio of mean fluorescence intensity (MFI) of CD56 at the NK92-THP-1 contact site to mean fluorescence intensity of CD56 at NK92 surface. **(B)** **p < 0.01, ****p < 0.0001.

The interaction of ICAM-1 with lymphocyte function-associated antigen-1 (LFA-1) on NK cells is important in the formation of the immune synapse (47). To verify whether decreased levels of ICAM-1 in the absence of STAT3 lead to impaired formation of NK-target cell conjugates, we incubated THP-1 with NK92 cells for 5 and 10 min and analyzed the number of doublets via flow cytometry. STAT3-deficient THP-1 cells barely formed any conjugates, while a significant increase in the NK-target cell doublets was observed in the control cell line after 5 and 10 min (**Figures 4A, B**). The same tendency was observed with another NK cell line, KHYG1 (**Supplementary Figure S4A**). In line, HEL cells lacking STAT3 formed significantly less conjugates upon 20 min incubation with NK92 or KHYG1 cells compared to the control HEL cell line (**Supplementary Figures S4B, C**). To further visualize the impaired NK-target adhesion, we imaged the conjugates upon 10 min incubation of THP-1 and NK92 cells using fluorescence microscopy. CD56-stained (red) NK92 cells (blue) appeared to attach to the target cells (green) to a higher degree in the presence of STAT3 than in its absence (**Figure 4C**). In line, CD56, which was shown to localize at the immune synapse in NK92 cells (48), was only enriched at the synapse area with STAT3-expressing target cells (**Figures 4C, D**). Thus, these data clearly demonstrate a reduced immune synapse formation between NK and AML cells in the absence of STAT3.

### 3.5 ICAM-1 deregulation strongly contributes to the STAT3^KO^ AML cells’ escape from NK cells

**Figure 5:**
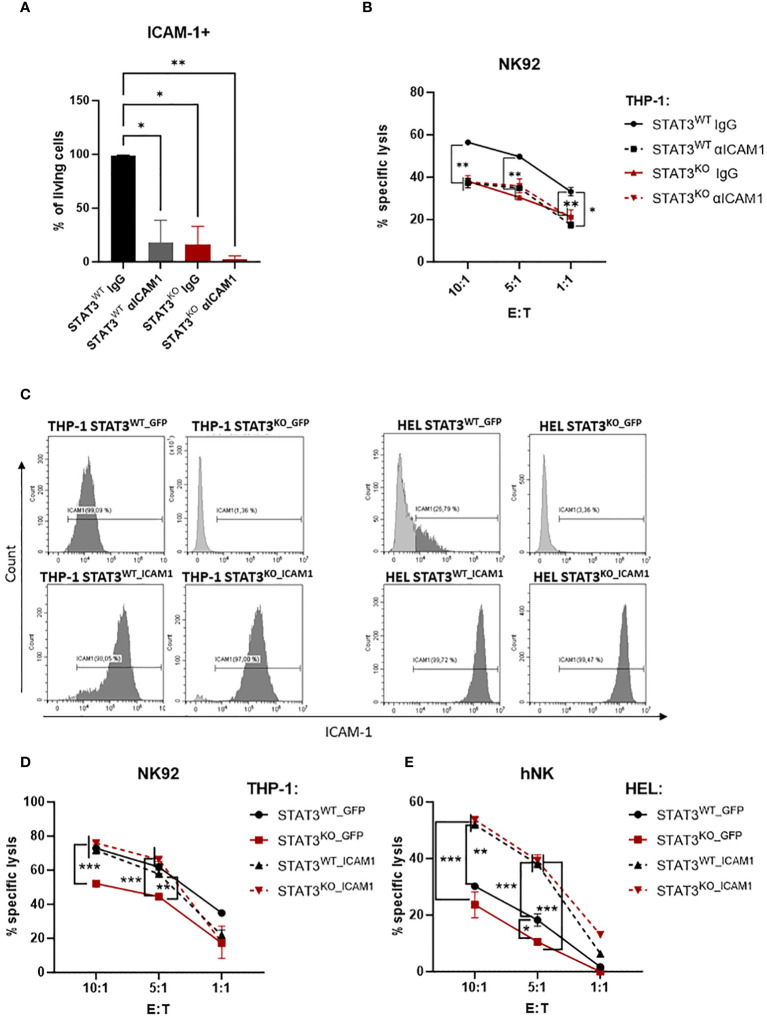
The impaired killing of STAT3-deficient AML cells is caused by low expression of ICAM-1. **(A)** THP-1 STAT3^WT^ and STAT3^KO8^ cells were incubated with isotype control (IgG) or blocking antibody against ICAM-1 followed by surface expression analysis of ICAM-1 via flow cytometry. Bar graphs show the mean percentage of ICAM-1^+^ cells +/- SD (n=2 per group from two independent experiments). Statistical analysis was performed using one-way ANOVA with Tukey post-test. **(B)** CFSE-stained THP-1 STAT3^WT^ and STAT3^KO8^ cells were preincubated with isotype control (IgG) or ICAM-1 blocking antibody and mixed at the indicated effector: target (E:T) ratios with NK92 cells for 4 h. The specific lysis of target cells was assessed by flow cytometry. One representative out of two independent experiments is shown. Symbols and bars represent the mean of technical duplicates +/- SD. Statistical analysis was performed using one-way ANOVA with Tukey post-test for each ratio. Parentheses indicate that the same significance level refers to all conditions covered. **(C)** THP-1 STAT3^WT^ and STAT3^KO8^ and HEL STAT3^WT^ and STAT3^KO8^ cells transduced with lentivirus encoding for GFP or ICAM-1 were analyzed for surface expression of ICAM-1 via flow cytometry. Representative flow cytometry plots out of 2 independent experiments. **(D, E)** CFSE-stained **(D)** THP-1 or **(E)** HEL STAT3^WT_GFP^, STAT3^KO_GFP^, STAT3^WT_ICAM1^ and STAT3^KO_ICAM1^ cells were mixed at indicated effector: target (E:T) ratios with **(D)** NK92 cells for 4 h or **(E)** primary human NK cells for 2 h. The specific lysis of target cells was assessed by flow cytometry. One representative out of two independent experiments is shown. Symbols and bars represent the mean of technical duplicates +/- SD. Statistical analysis was performed using one-way ANOVA with Tukey post-test for each ratio. Parentheses indicate that the same significance level refers to all conditions covered. **(A, B, D, E)** *p < 0.05, **p < 0.01, ***p < 0.001.

To confirm that the impaired killing was due to the strongly reduced expression of ICAM-1 on the surface of STAT3-deficient AML cells, we pre-incubated THP-1 STAT3^WT^ and STAT3^KO^ cells with either IgG control antibody or ICAM-1 blocking antibody and assessed the killing capacity of NK92 cells. ICAM-1 blocking efficiency was confirmed via flow cytometry (**Figure 5A**). Indeed, the killing of the THP-1 control cell line was impaired upon blocking of ICAM-1 to the levels comparable to STAT3-deficient cells (**Figure 5B**). Importantly, blockage of ICAM-1 did not further impair the cytotoxicity against STAT3^KO^ cells (**Figure 5B**), showing that the inefficient killing is attributable to reduced ICAM-1 expression. In line with only a fraction of HEL cells expressing ICAM-1, blocking of ICAM-1 in HEL cells resulted in a tendency for reduced killing of HEL STAT3^WT^ cells while HEL STAT3^KO^ cell killing was not affected (**Supplementary Figure S4D**).

To further confirm the crucial role of ICAM-1 in the escape of STAT3-deficient AML cells we lentivirally transduced THP-1 and HEL STAT3^WT^ and STAT3^KO^ cells with a vector encoding for *GFP* only (STAT3^WT_GFP^ or STAT3^KO_GFP^) or human *ICAM1* and *GFP* (STAT3^WT_ICAM1^ or STAT3^KO_ICAM1^). In GFP expressing cell lines, the ICAM-1 expression pattern remained unchanged to the untransduced cell lines with STAT3^KO_GFP^ cells expressing barely any ICAM-1 (**Figure 5C** upper panels). All *ICAM1*-transduced cell lines expressed high ICAM-1 on the cell surface proving successful overexpression (**Figure 5C** lower panels). The expression of GFP did not affect the previously observed killing pattern (**Figures 2A–D**) and both THP-1 and HEL STAT3^KO_GFP^ cells were killed significantly less efficiently than the respective controls (**Figures 5D, E**). Overexpression of ICAM-1 rescued recognition of STAT3-deficient cells and resulted in comparable killing levels of STAT3^KO_ICAM1^ and STAT3^WT_ ICAM1^ in both cell lines (**Figures 5D, E**). As expected from the fact that only a fraction of HEL STAT3^WT^ cells express ICAM-1 (**Figures 3C, D**), ICAM-1 overexpression significantly increased killing already in HEL STAT3^WT^ cells (**Figure 5E**). In summary, our data demonstrate that STAT3-deficient AML cells escape NK-cell recognition via downregulation of ICAM-1 thereby impairing their adhesion to NK cells.

### 3.6 *STAT3* correlates with *ICAM1* expression in AML patient samples

**Figure 6:**
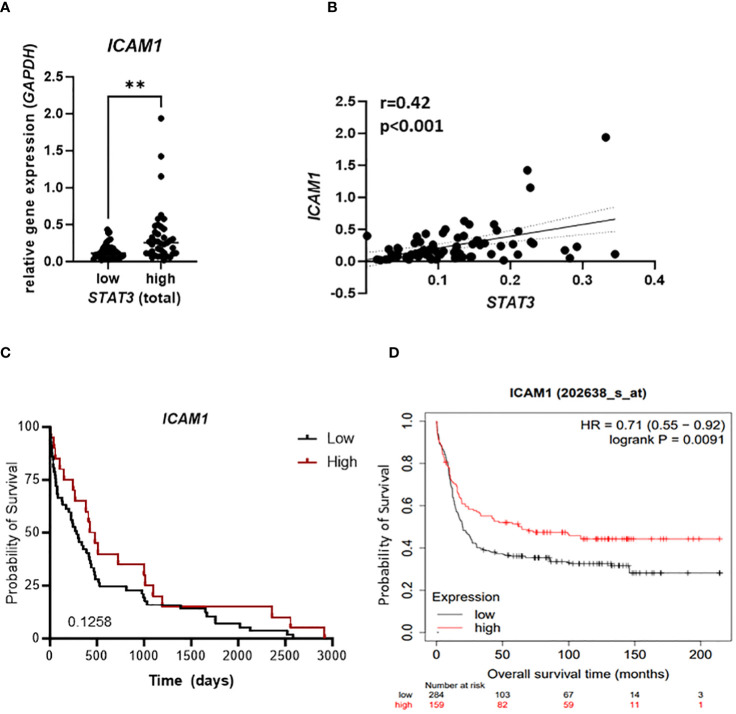
*STAT3* correlates with *ICAM1* expression in AML patient samples. **(A–C)** 79 AML patient samples were analyzed for expression of *STAT3* and *ICAM1* using RT-qPCR and **(A)** *ICAM1* expression was compared between *STAT3* high and *STAT3* low group. Statistical analysis was performed using unpaired t-test; **p < 0.01. **(B)** The correlation between *STAT3* and *ICAM1* expression was analyzed using Pearson test. **(C)** Kaplan-Meier plot shows the probability of survival of AML patients expressing high or low *ICAM1.* **(D)** Kaplan Meier plot shows the probability of survival of AML patients from publicly available datasets (kmplot.com) stratified into *ICAM1* high and low groups. **(C, D)** Statistical analysis was performed using log-rank (Mantel-Cox) test.

To evaluate clinical relevance of our findings we analyzed samples from 79 newly diagnosed AML patients for the mRNA expression of *STAT3* and *ICAM1*. Patients expressing high levels of *STAT3* showed significantly higher expression of *ICAM1* (**Figure 6A**). In line, we observed a positive correlation of *STAT3* and *ICAM1* in our patient cohort (**Figure 6B**) as well as in a publicly available dataset [**Supplementary Figure S5A**; (40)]. Importantly, no significant correlation was observed between *ICAM1* and *STAT1* expression (**Supplementary Figure S5B**), which has previously been implicated in driving transcription of *ICAM1* (49). Interestingly, among the top five enriched hallmarks in GSEA of *ICAM1* correlating genes in the TCGA AML database, we identified the IL6_JAK_STAT3_SIGNALING hallmark pointing towards a correlation of *ICAM1* with not only *STAT3* expression, but also STAT3 activity (**Supplementary Figure S5C**). These data strengthen our findings on a STAT3-dependent positive regulation of ICAM-1 in AML. In our patient cohort we also observed a trend towards improved survival of patients with high *ICAM1* expression (**Figure 6C**), which could be further validated in the publicly available dataset, where this correlation reached statistical significance [**Figure 6D**; (40)]. Furthermore, we explored clinical features (age, gender, karyotype) associated with elevated levels of *ICAM1*. In a multivariate analysis including age and karyotype but not gender, *ICAM1* expression retained significance (**Supplementary Figure S5D**).

In summary, we provide the first evidence for an unexpected, NK cell-sensitizing effect of STAT3 in AML mediated by ICAM-1-dependent immune synapse formation. In light of our data, ICAM-1 might become an interesting candidate for a biomarker predicting responses to NK-cell therapy in AML.

## 4 Discussion

In this study we uncovered a novel role of STAT3 in sensitizing AML cells to NK cell-mediated lysis providing first evidence for the STAT3/ICAM-1 axis in AML cells as a prerequisite for efficient immune synapse formation with NK cells.

Targeting STAT3 has been shown to induce immunostimulatory effects in different cancer types including AML (24, 25, 50, 51), but the role of STAT3 in AML cell susceptibility to NK cells has not been thoroughly studied. Although inhibition of the JAK/STAT3 pathway resulted in increased expression of NKG2D ligands and thereby increased killing by NK cells for some leukemic cell lines, the effects cannot be solely attributed to STAT3 (29, 30, 52). In contrast, loss of STAT3 in B cell lymphoma cells decreased killing by NK cells *in vitro* and *in vivo* (53). This is in line with our findings that STAT3-deficient AML cell lines escaped recognition by NK cells. We observed this effect with primary human NK cells and two distinct NK cell lines (NK92 and KHYG1) suggesting a common escape mechanism. We could not detect any impact of the STAT3^KO^ on the levels of NKp30 ligand B7-H6, which has been previously indicated to impact on killing of THP-1 cells (45). Interestingly, MICA/B and ULBP2/5/6 were downregulated in THP-1 STAT3^KO^ cells, while the levels in STAT3^KO^ HEL cells remained unchanged. As NCR-dependent activation was shown to synergize with NKG2D-dependent signals in the NK-killing of THP-1 cells (16), we cannot exclude that NKG2D ligand downregulation contributes to the observed effects, albeit only in THP-1 and not HEL cells. However, in both cell lines a clear pattern of decreased mRNA expression of the adhesion molecule *ICAM1* was observed in the absence of STAT3 with strong downregulation of ICAM-1 on the surface. ICAM-1 seems to be the predominant mechanism responsible for myeloid leukemia cell killing as a CRISPR screen in the K562 cell line identified ICAM-1 perturbation with the highest score of NK92 evasion mechanisms (54).

The LFA-1-ICAM-1 interaction has an important role in the formation of the immune synapse between an NK cell and a target cell (47, 55). The binding of ICAM-1 to LFA-1 induces granule polarization and is therefore a prerequisite for efficient degranulation (56, 57). Indeed, we observed that STAT3^KO^ AML cells induced significantly less degranulation, and failed to form conjugates with NK cells as efficiently as STAT3^WT^ cells. Blocking antibodies against ICAM-1 reduced the killing of STAT3^WT^ cells but had no effect on the cytotoxicity against STAT3^KO^ cells. Most importantly, overexpression of ICAM-1 in STAT3-deficient AML cells could rescue the decreased NK-cell susceptibility to the levels of STAT3^WT^ cells confirming the impaired ICAM-1-dependent adhesion to NK cells as an escape mechanism of STAT3^KO^ cells. The LFA-1-ICAM-1 interaction was recently shown to have a key role in T-cell cytotoxicity against AML (58). Therefore, this mechanism might not only apply to the escape from NK cells but also contribute to the impaired conjugation of AML cells with cytotoxic T cells suggesting a general relevance of our findings.

Previously, the induction of ICAM-1 expression on the surface of AML cells by cytokines such as GM-CSF, IFNγ or TNFα was associated with improved NK-cell cytotoxicity but the dependence on STAT3 has not been shown in this context (59, 60). In other cell types, including endothelial cells, STAT3 has been described to directly regulate ICAM-1 in response to IL-6 (49, 61–63). In AML, STAT3 from the nuclear extract of patient leukemia cells stimulated with IL-6 binds the probe of an IL-6 responsive element derived from the *ICAM1* promoter (64). Our data also point toward direct regulation of *ICAM1* by STAT3 in AML cell lines. Interestingly, a strong STAT3 dependence of ICAM-1 expression was not only present in the HEL cell line harboring a constitutive STAT3 phosphorylation due to the activating mutation of JAK2 (35, 65), but also in THP-1 cells where it might be dependent on low levels of autocrine STAT3 activating cytokines (66, 67). In line, we detected a positive correlation of *STAT3* and *ICAM1* in two different AML patient cohorts. However, also several other transcription factors have been implicated in driving ICAM-1 expression including STAT1 (49, 68, 69). Our data suggest that STAT1 does not suffice to drive ICAM-1 expression in AML cell lines. In line, *STAT1* did not correlate with *ICAM1* expression in our patient cohort pointing towards a predominant role of STAT3 in regulating ICAM-1 in this disease.

Previous reports in solid tumors correlated high *ICAM1* expression with worse prognosis and invasive potential of the tumor cells (70, 71). In contrast, in AML high *ICAM1* expression correlated with better survival in a publicly available dataset. It is attractive to speculate that these patients exhibit a better immune response against their minimal residual disease but the results might also be affected by other functions of ICAM-1 including cell migration and trafficking (69). Moreover, NK cells of AML patients are defective in their function (7). However, in the context of emerging NK cell-based therapies, it remains a very attractive approach to induce ICAM-1 expression on the surface of leukemic cells to increase their visibility to NK cells. Xiao et al., show that AML cells express lower *ICAM1* than healthy controls and this effect can be reversed by decitabine treatment. In line, decitabine increased the killing by NK cells but ICAM-1 upregulation seemed not to be the only mechanism behind it (72). Also, metformin has been discovered to induce NKG2D ligands and ICAM-1 expression in AML cell lines paralleled by increased killing by NK cells *in vitro* (73). However, a more robust screening approach will be necessary to identify ICAM-1 inducing and NK-cell sensitizing drugs.

In summary, we uncovered a crucial role of STAT3 in AML cell escape from NK cells and identified the STAT3/ICAM-1 axis as one of the key mechanisms of NK-cell mediated leukemic cell lysis. Thus, we propose STAT3/ICAM-1 as a novel biomarker set for susceptibility to NK cells, which could be used in the future to screen patients with AML before NK cell-based therapy.

## Data availability statement

The raw data supporting the conclusions of this article will be made available by the authors, without undue reservation.

## Ethics statement

The animal study was approved by Ethics and Animal Welfare Committee of the Medical University of Vienna. The study was granted approval by the Scientific Integrity and Ethics Committee at Karl Landsteiner University of Health Sciences in Krems, Austria, and adhered to the principles of the Declaration of Helsinki. The study was conducted in accordance with the local legislation and institutional requirements.

## Author contributions

AW-S: Writing – original draft, Conceptualization, Funding acquisition, Investigation, Methodology, Data curation, Formal analysis. CD: Investigation, Writing – review & editing, Data curation. BZ: Conceptualization, Investigation, Methodology, Writing – review & editing, Formal analysis. LH: Investigation, Writing – review & editing, Formal analysis. SE: Investigation, Writing – review & editing, Formal analysis. TH: Investigation, Writing – review & editing. SW: Investigation, Writing – review & editing. KH: Investigation, Writing – review & editing. MH: Writing – review & editing, Data curation, Methodology. SS: Resources, Writing – review & editing. SD: Resources, Writing – review & editing. EC: Funding acquisition, Resources, Writing – review & editing. GO: Writing – review & editing, Methodology, Supervision, Resources. BG: Writing – review & editing, Methodology, Resources. EP: Funding acquisition, Methodology, Resources, Writing – review & editing. HS: Resources, Writing – review & editing. DS: Conceptualization, Funding acquisition, Resources, Supervision, Writing – review & editing.
