# Supplementary material for: STAT3 in acute myeloid leukemia facilitates natural killer cell-mediated surveillance
Source: Front Immunol. 2024 Jul 5;15:1374068. doi: 10.3389/fimmu.2024.1374068 (PMC11257888; doi:10.3389/fimmu.2024.1374068)
Supplement: Supplementary file 1 [file DataSheet_1.pdf]

Figure S1

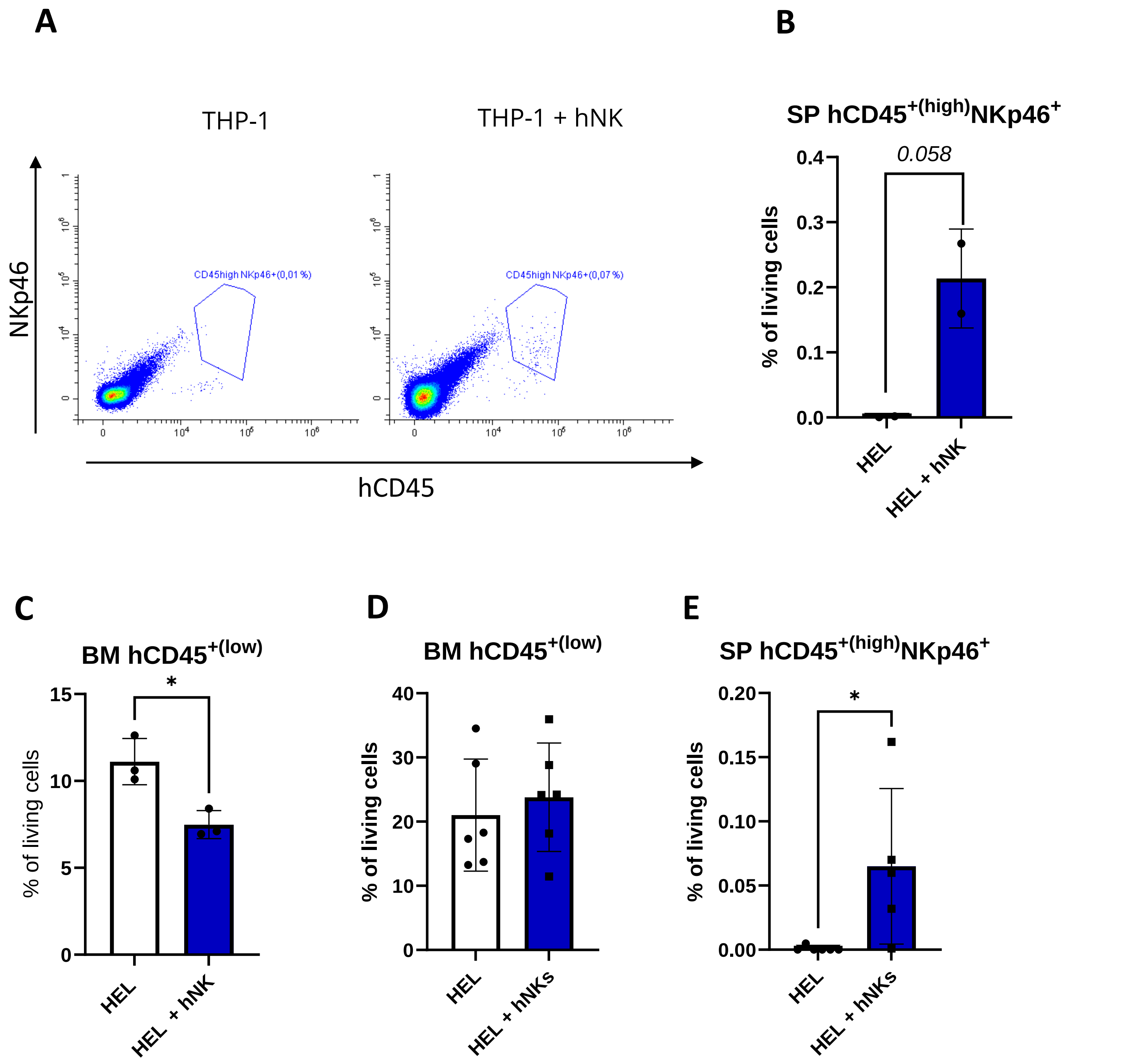

Figure S1. THP-1 and HEL cell line xenografts are killed by NK cells *in vivo*. (A-E) NSG-Tg(Hu-IL15) mice were injected with $0.5x10^6$ **(A)** THP-1 cells or **(B-E)** HEL cells *i.v.*. In addition, the mice were injected with 10^6 human NK cells 24 h prior to leukemic cell inoculation or left untreated. **(A)** NK cells (hCD45$^{+(high)}$NKp46$^+$) in the splenic single cell suspensions of THP-1-injected animals on day 28 post injection were analyzed by flow cytometry. **(B,C): (B)** NK cells (hCD45$^{+(high)}$NKp46$^+$) in the splenic single cell suspensions or **(C)** leukemic cells (hCD45^{low}) in bone marrow of HEL-injected animals were analyzed by flow cytometry on day 28 post injection. **(D, E): (D)** leukemic cells (hCD45^{low}) in bone marrow or **(E)** NK cells (hCD45$^{+(high)}$NKp46$^+$) in the splenic single cell suspensions of HEL-injected animals were analyzed by flow cytometry at the disease endpoint. **(A)** Representative flow cytometry plots. **(B, C)** Bar graphs represent mean +/- SD from 1 experiment (n=2-3 per group); **(D, E)** Bar graphs represent mean +/- SD from 2 independent experiments (n=6 per group); **(B, C, E)** Statistical analysis was performed using unpaired t-test . * $p<0.05$;

Figure S2

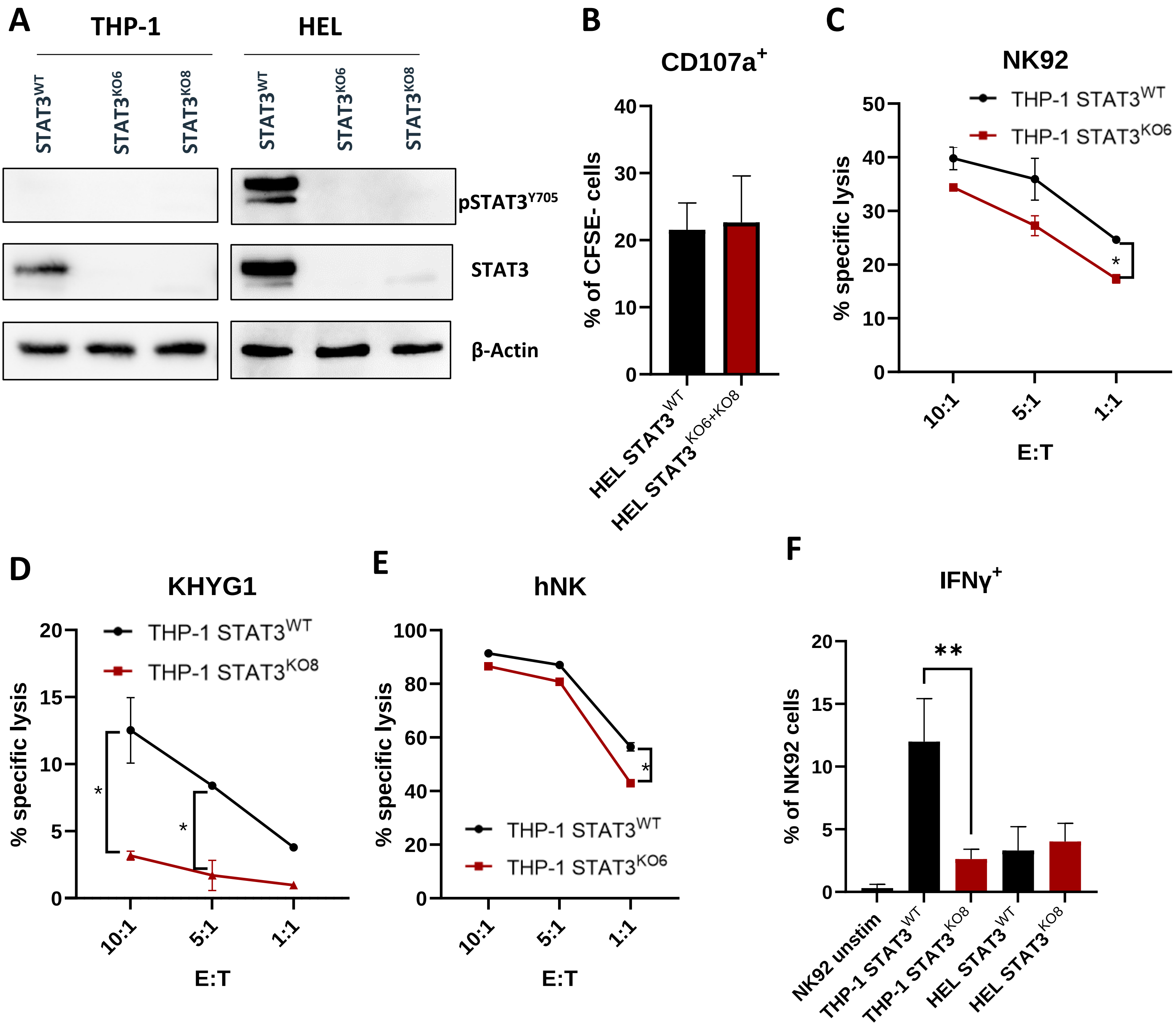

Figure S2. STAT3-deficient AML cells escape NK cell recognition. (A) Levels of pSTAT3, STAT3 and β-actin were assessed by Western blot in cell lysates of THP-1 and HEL STAT3^{WT}, STAT3^{KO6} and STAT3^{KO8} and a representative blot out of three independent experiments is shown. **(B)** CFSE stained HEL STAT3^{WT} and STAT3^{KO6} and STAT3^{KO8} cells were mixed at 1:1 ratio with human primary NK cells from three different healthy donors and the percentage of CD107a+ NK cells (CFSE-; in contrast to CFSE+ target cells) was analyzed by flow cytometry. Bar graphs represent mean +/- SD of n= 4 per group (3 donors, 1-2 experiments per donor).**(C, E)** CFSE-stained THP-1 STAT3^{WT} and STAT3^{KO6} cells were mixed at indicated effector : target (E:T) ratios with **(C)** NK92 for 4 h or **(E)** expanded primary human NK cells for 2 h. **(D)** CFSE-stained THP-1 STAT3^{WT} and STAT3^{KO8} cells were mixed at indicated effector : target (E:T) ratios with KHYG1 for 4 h. **(C-E)** Symbols and bars represent mean of technical duplicates +/- SD. Statistical analysis for each ratio was performed using unpaired t-test. **(F)** NK92 cells were stained for intracellular IFNγ upon incubation with STAT3^{WT} or STAT3^{KO8} THP-1 or HEL cell line. Bar graphs represent mean +/- SD of n= 4 per group from 2 independent experiments. Statistical analysis for each cell line separately was performed using unpaired t-test . * $p < 0.05$; ** $p < 0.01$

Figure S3

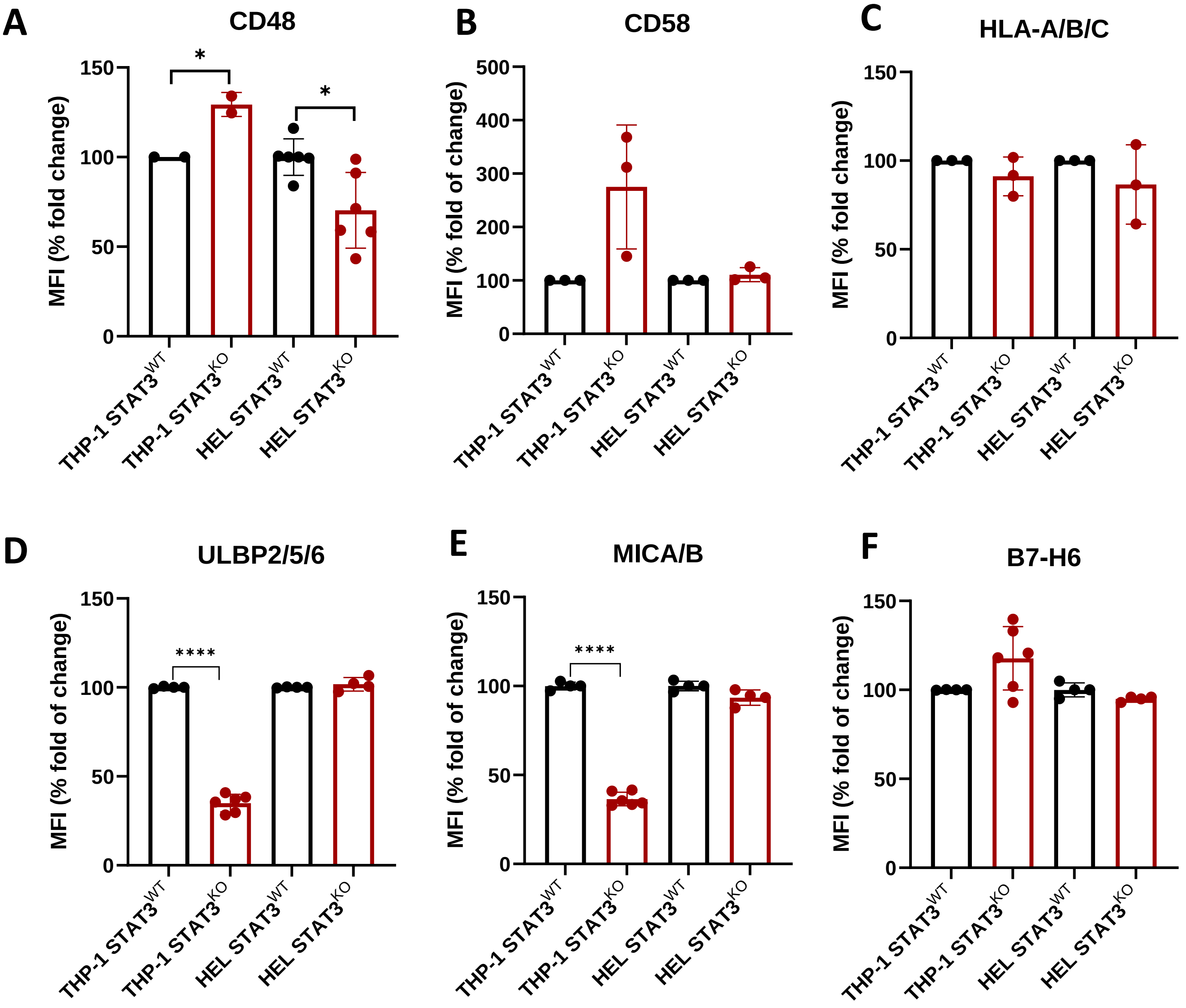

Figure S3. Surface expression of NK cell ligands. (A, B) STAT3^{WT} and STAT3^{KO8} THP-1 and HEL were analyzed for surface expression of CD48, CD58, HLA-A/B/C, ULBP2/5/6, MICA/B, B7-H6 via flow cytometry. Bar graphs showing mean fluorescence intensity (MFI) as percentage of fold change of **(A)** CD48 **(B)** CD58, **(C)** HLA-A/B/C, **(D)** ULBP2/5/6, **(E)** MICA/B and **(F)** B7-H6 +/- SD are shown for each cell line (n=3-6 per group from 2-3 independent experiments). Statistical analysis was performed using unpaired t test for THP-1 and HEL cells separately; * $p < 0.05$; ****<0.0001

Figure S4

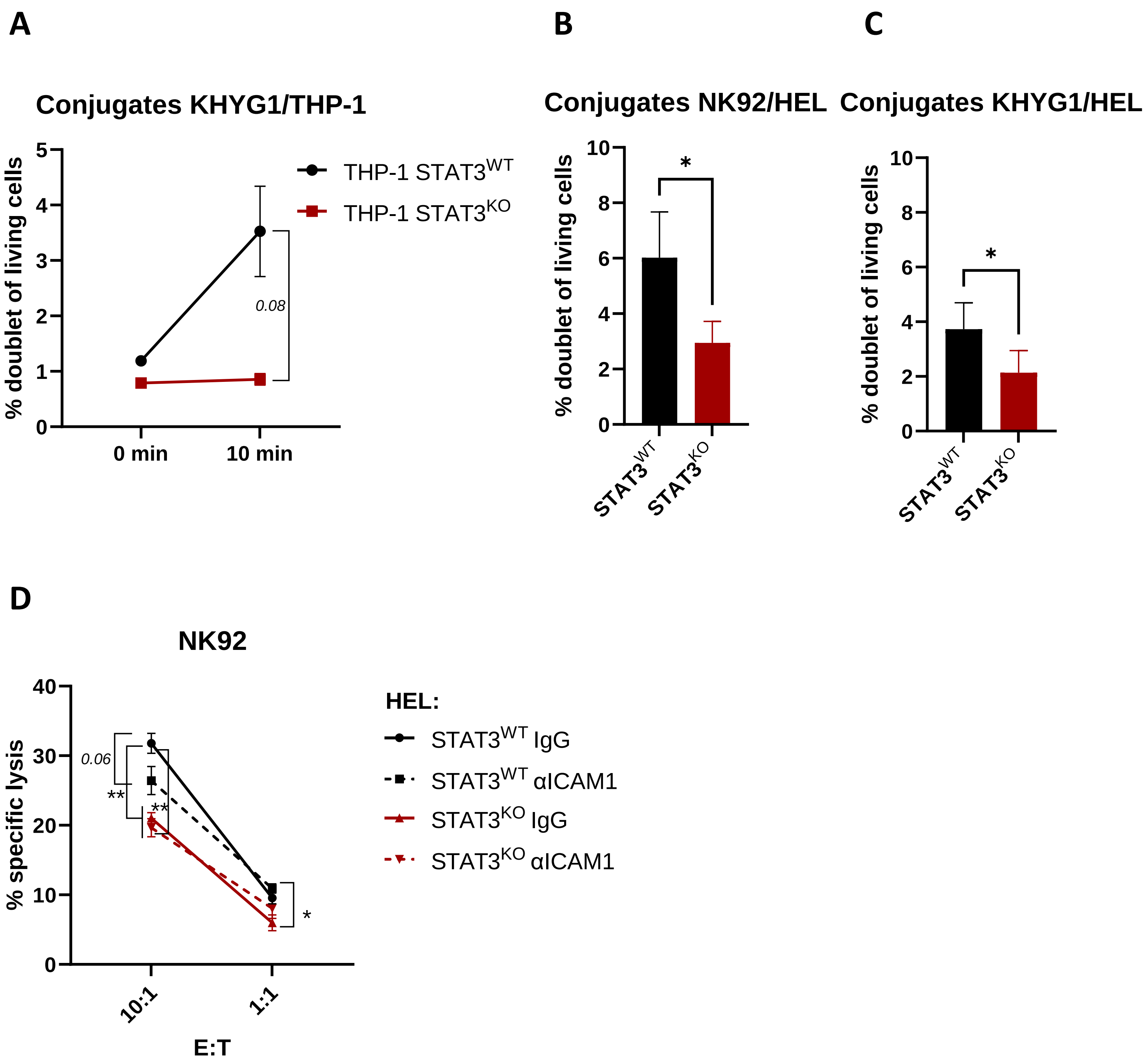

Figure S4. STAT3-deficient AML cells form fewer synapses. (A) THP-1 $STAT3^{WT}$ and $STAT3^{KO8}$ or **(B, C)** HEL $STAT3^{WT}$ and $STAT3^{KO8}$ were incubated with **(A, C)** KHYG1 or **(B)** NK92 cells at 1:1 ratio and the amount of NK-target doublets was analyzed after **(A)** 10min or **(B, C)** 20 min. **(A)** Representative time course out of two independent experiments. For timepoint 10 min dots represent the mean percentage of doublets +/- SD (n=2). **(B, C)** Bar graphs represent the mean percentage of doublets +/- SD (n=2-4 per group from one to two independent experiment); (A-C) Statistical analysis was performed using unpaired t-test. * $p < 0.05$. **(D)** CFSE-stained HEL $STAT3^{WT}$ and $STAT3^{KO8}$ cells were preincubated with isotype control (IgG) or ICAM-1 blocking antibody and mixed at indicated effector : target (E:T) ratios with NK92 cells for 4 h. The specific lysis of target cells was assessed by flow cytometry. One representative out of two independent experiments is shown. Symbols and bars represent the mean of technical duplicates +/- SD. Statistical analysis was performed using one-way ANOVA with Tukey post-test for each ratio. Vertical lines indicate that the same significance level refers to all conditions covered by the line. ** p<0.05; **p<0.01

Figure S5

A

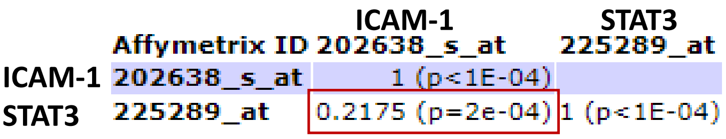

Pearson correlation coefficient (r)

	Affymetrix ID	ICAM-1 202638_s_at	STAT3 225289_at
ICAM-1	202638_s_at	1 (p<1E-04)	
STAT3	225289_at	0.2175 (p=2e-04)	1 (p<1E-04)

B

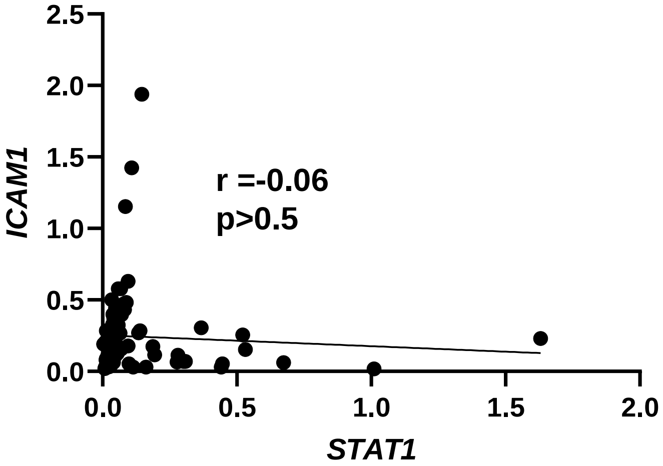

C

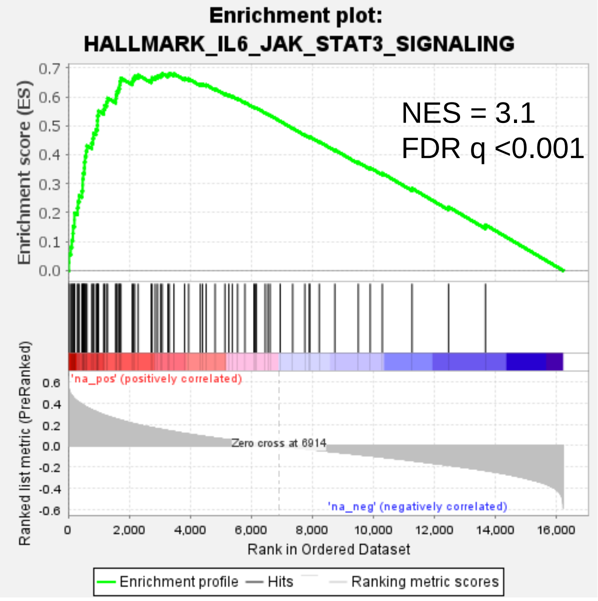

D

	Multivariate analysis		
	Hazard ratio	*95% CI*	*p value*
**Age**	1.01	1.004-1.024	0.004
**Gender**	0.98	0.75-1.21	n.s.
**Karyotype**	2.08	1.90-2.26	7.1E-07
	**Univariate analysis**		
	Hazard ratio	*95% CI*	*p value*
**Age**	1.03	1.022-1.030	1.2E-06
**Gender**	0.96	0.79-1.13	n.s.
**Karyotype**	1.85	1.71-1.99	6.7E-07

Figure S5. *STAT3* correlates with *ICAM1* in AML patients. (A, B) The correlation between **(A)** *STAT3* and *ICAM1or* **(B)** *STAT1* and *ICAM1* expression was analyzed using Pearson test in **(A)** publicly available dataset (kmplot.com) and **(B)** in a cohort of 79 AML patients as described in Fig. 5. **(C)** Enrichment plot of the hallmark IL6_JAK_STAT3_SIGNALING. Red and blue colors represent, respectively, positive and negative Spearman's correlations with *ICAM1*. Analysis was performed on normalized gene expression RNA-seq data of human AML patients (TCGA-LAML) from The Cancer Genome Atlas (TCGA). Normalized enrichment score (NES) and false discovery rate (FDR) q value are indicated. **(D)** Uni- and multivariate Cox regression analysis was performed to correlate survival and *ICAM1* expression, age, gender, karyotype. The multivariate analysis was performed by pairing *ICAM1* with the clinical variables and the hazard ratio, 95% confidence interval (CI) and p value are shown for each variable.

Table S1

**Total number of patients**	**79**
Males	50 (63.3%)
Females	29 (36.7%)
**Age (years)**	
Median	66
Range	23-89
**WBC at diagnosis (10^9/l)**	
Median	6
Range	0-388
**BM blasts at diagnosis (%)**	
Median	68
Range	15-100
**Pathogenic/likely pathogenic mutations (number of samples)**	
KRAS	12
NRAS	14
RUNX1	13
IDH2	8
WT1	2
ASXL1	14
TET2	24
DNMT3A	13
NPM1	11
CEBPA	13
TP53	17
FLT3 / FLT3-IDH	0
Other Mutations (IDH1, CRSF2, STAG2, KIT, etc.)	48
**Therapy**	
Intensive incl. allogeneic hematopoietic stem cell transplantation	45
Non-intensive	28
Best supportive care	6

Table S1. Characteristics of 79 AML patients.

Table S2

GAPDH Fw	TCTCCTCTGACTTCAACAGCG
GAPDH Rev	ACCACCCTGTTGCTGTAGCC
ULBP1 Fw	TTTCCTTAAAGGGCAACTGCT
ULBP1 Rev	AGGAACTGCCAAGATCCTCT
MICA Fw	GAATCCGGCGTAGTCCTGAG
MICA Rev	TCCGGGGATAGAAGCTGGAA
B7H6 Fw	TTTTCCATTCATTGGTGGCCT
B7H6 Rev	TGCCCGAGTGCAAAAGAATATG
HLA_A Fw	AGATACACCTGCCATGTGCAGC
HLA_A Rev	GATCACAGCTCCAAGGAGAACC
ICAM1 Fw	AGCGGCTGACGTGTGCAGTAA T
ICAM1 Rev	TCTGAGACCTCTGGCTTCGTCA
CD48 Fw	GCTTGAAACCACCCTTATGCCAC
CD48 Rev	CGTGACCACTAGCCAACTTGCA
CD58 Fw	GACACTGTGTCAGGTAGCCTCA
CD58 Rev	GCACAAGTTAGTGTGGGAGATGG
STAT3 Fw	CTGACCCAGGTAGCGCTGCCCCATACC
STAT3 Rev	TCACAATGGGGGAGGTAGCGCACTCCG

Table S2. Primer list used for RT-qPCR analysis.
